# HnRNP A1/A2 and SF2/ASF Regulate Alternative Splicing of Interferon Regulatory Factor-3 and Affect Immunomodulatory Functions in Human Non-Small Cell Lung Cancer Cells

**DOI:** 10.1371/journal.pone.0062729

**Published:** 2013-04-29

**Authors:** Rong Guo, Yong Li, Jinying Ning, Dan Sun, Lianjun Lin, Xinmin Liu

**Affiliations:** 1 Department of Geriatrics, Peking University First Hospital, Beijing, China; 2 Key Laboratory of Carcinogenesis and Translational Research (Ministry of Education), Department of Laboratory Animal, Peking University Cancer Hospital, Beijing Cancer Hospital, Beijing Institute for Cancer Research, Beijing, China; 3 Department of Cell Biology, Crown Bioscience Incorporation (Beijing), Beijing, China; University of Nebraska - Lincoln, United States of America

## Abstract

Heterogeneous nuclear ribonucleoparticule A1/A2 (hnRNP A1/A2) and splicing factor 2/alternative splicing factor (SF2/ASF) are pivotal for precursor messenger RNA (pre-mRNA) splicing. Interferon regulatory factor-3 (IRF-3) plays critical roles in host defense against viral and microbial infection. Truncated IRF-3 proteins resulting from alternative splicing have been identified and characterized as functional antagonists to full-length IRF-3. In this study, we examined the molecular mechanism for splicing regulation of IRF-3 pre-mRNA and first reported the regulatory effect of hnRNP A1/A2 and SF2/ASF on IRF-3 splicing and activation. RNA interference-mediated depletion of hnRNP A1/A2 or SF2/ASF in human non-small cell lung cancer (NSCLC) cells increased exclusion of exons 2 and 3 of IRF-3 gene and reduced expression levels of IRF-3 protein and IRF-3 downstream effector molecules interferon-beta and CXCL10/IP-10. In addition, direct binding of hnRNP A1 and SF2/ASF to specific binding motifs in IRF-3 intron 1 was confirmed by RNA electrophoretic mobility shift assay. Subsequent minigene splicing assay showed that IRF-3 minigenes with mutated hnRNPA 1/A2 or SF2/ASF binding motifs increased exclusion of exons 2 and 3. Moreover, knockdown of hnRNP A1/A2 or SF2/ASF in NSCLC cells reinforced phytohemagglutinin-induced tumor necrosis factor-alpha release by peripheral blood mononuclear cells (PBMC) but suppressed that of interleukin-10 in NSCLC/PBMC co-cultures. Taken together, our results suggest that specific knockdown for hnRNP A1/A2 or SF2/ASF increase exclusion of exons 2 and 3 of IRF-3 pre-mRNA and influence immunomodulatory functions of human NSCLC cells.

## Introduction

Alternative precursor messenger RNA (pre-mRNA) splicing is an important posttranscriptional mechanism by which cells can generate a diverse repertoire of protein isoforms from a more limited number of genes [1]. It is estimated that the majority of human multi-exon genes are alternatively spliced [2]. Alternative splicing plays important roles in development, physiology, and disease and the process of removing introns selectively and joining of residual exons is subject to precise regulation and is often disturbed in inflammatory disorders and cancers [3]–[6]. Numerous researches have proved that some RNA-binding proteins may participate in regulation of inflammatory process and tumorigenesis by regulating splicing or mRNA stability of inflammation- and tumor-related genes [4], [6]–[8]. Two nuclear RNA-binding protein families, the family of heterogeneous nuclear ribonucleoproteins (hnRNP) and the family of serine/arginine-rich proteins (SR), play pivotal roles in regulation of alternative splicing and mRNA stability. The hnRNP family contains at least twenty members and mainly binds to sequences called splicing silencers, located in exons (ESSs, exonic splicing silencers) or introns (ISSs, intronic splicing silencers), to promote exon exclusion and act as splicing repressors [9]. The most abundant and best characterized proteins of this group are hnRNP A1 and hnRNP A2, which share a high degree of sequence homology and functional homology [10]. Increasing evidences have demonstrated that hnRNP A1 and hnRNP A2 are over-expressed in various kinds of tumors and serve as early tumor biomarkers [7], [11]–[13]. HnRNP U, as another hnRNP family member, has been reported to enhance TLR-induced proinflammatory cytokine production by stabilizing mRNAs in macrophages [14]. The family of SR proteins, another regulator for alternative splicing, also includes more than twenty members. These proteins bind to splicing enhancers which locate in exons (ESEs, exonic splicing enhancers) or introns (ISEs, intronic splicing enhancers), and predominantly function as antagonists of hnRNP proteins [15]. However, a number of studies have also revealed that SR proteins regulate exon skipping events and different SR proteins show opposite activities in promoting exon inclusion or skipping on the same genes [16], [17]. Splicing factor 2/alternative splicing factor (SF2/ASF), as the best characterized member of the SR family, has been reported to be up-regulated in multiple human cancers, including lung cancer and cervical cancer, and plays important roles in the establishment and maintenance of cell transformation [8], [18]–[20]. Recent research also revealed that SF2/ASF mediated IL-17-induced mRNA stability of chemokine CXCL1 in human cervical cancer cells [21].

The continuously growing interferon regulatory factor (IRF) family includes transcriptional activators and repressors which regulate gene expression critical to immune response, hematopoiesis, and cell survival [22]–[24]. IRF-3 is unique among IRF family members in that it is a key direct transducer of viral double-stranded RNA and bacterial lipopolysaccharide-mediated signaling [25], [26]. IRF-3 serves as an essential transcriptional activator for type I interferons (IFNα/β), a subset of interferon-stimulated genes as well as some chemokine genes such as RANTES and CXCL10/IP-10 and plays critical roles both in the innate immune response against viral infection and the subsequent activation of adaptive immunity [27]–[31]. The IRF-3 gene consists of 8 exons and 7 introns and encodes a 427-amino acid protein. IRF-3 is a phosphoprotein and consists of an N-terminal DNA-binding domain (DBD) (amino acids 1 to 110), a C-terminal IRF-associated domain (IAD, amino acids 198 to 374), and a transactivation domain (TAD, amino acids 134 to 394) [32]. With its critical roles in host defense, the activity of IRF-3 is strictly controlled. IRF-3 is widely expressed and is found predominantly in an inactive cytoplasmic form. Following infection, virus or double-stranded RNA induces phosphorylation of C-terminal serine/threonine residues and leads to a conformational change in IRF-3 with exposure of both the DBD and IAD domains, which further results in homo- or heterodimerization, cytoplasm-to-nucleus translocation, association with CBP/p300 coactivators, and transcriptional activation of multiple target genes [33], [34].

Structural integrity is essential for IRF-3 to perform its regular function in myriad cellular pathways, as has been demonstrated by functional changes resulting from alternative splicing of IRF-3 pre-mRNA. IRF-3a is the first characterized IRF-3 splicing isoform and its original exon 2 found in IRF-3 is replaced by intron 2 [35]. IRF-3a protein lacks a portion of the N-terminal DBD of IRF-3 and is unable to bind to classical IRF binding elements, such as IFN-stimulated response elements (ISREs). However, IRF-3a with an intact IAD domain has been shown to form a heterodimer with IRF-3 following viral infection and inhibit IRF-3 transcriptional activity [36]. Recently, we identified five novel splicing variants of IRF-3, referred to as IRF-3b, -3c, -3d, -3e, and -3f, and revealed that these splicing variants are generated by deletion of exons 2, 3, or 6 or some combination thereof [37]. These novel splicing isoforms were predicated to lose the entire region of the DBD domain (IRF-3b, -3c, -3d, and -3f) or lose portions of the TAD and IAD domains (IRF-3b, -3d, and -3e). In addition, we also demonstrated that these novel splicing variants were expressed in various kinds of human cells and tissues and appeared to function as negative modulators of IRF-3 and reduce the transactivation of the IFNβ promoter to different extents. These established structural and functional changes owing to alternative splicing warrant further investigation of the molecular mechanism for splicing regulation of IRF-3 pre-mRNA.

In the present study, RNA interference-mediated depletion of hnRNP A1/A2 or SF2/ASF in human non-small cell lung cancer (NSCLC) cells increased exclusion of exons 2 and 3 of IRF-3 pre-mRNA. By using sequence analysis and programs such as ESE Finder, we identified hnRNP A1/A2 binding site (UAGGGA) and SF2/ASF binding site (GGAAGGA) in IRF-3 intron 1. Subsequent electrophoretic mobility shift assay (EMSA) using wild type (wt) RNA probe or RNA with mutation in these binding sites and mutant IRF-3 minigene splicing assay confirmed the presence of hnRNP A1/A2 and SF2/ASF binding motifs in IRF-3 intron 1. Moreover, the influence of change of IRF-3 splicing pattern on expression of its downstream target genes and on cytokine production in NSCLC/peripheral blood mononuclear cell (PBMC) co-cultures was also evaluated.

## Materials and Methods

### Cell Culture and RNA Interference

Human NSCLC cell lines A549 and Calu-6 were obtained from ATCC (Manassas, VA). Cells were cultured in RPMI 1640 medium supplemented with 10% heat-inactivated fetal bovine serum (FBS) (Invitrogen, Carlsbad, CA), 100 U/ml penicillin, and 100 µg/ml streptomycin at 37°C with 5% CO_2_.

Small interference RNAs (siRNAs) targeting hnRNP A1, hnRNP A2, SF2/ASF, or polypyrimidine tract binding protein (PTB or hnRNP I) mRNA were selected and siRNA transfections were performed as described [7], [38]. Due to functional redundancy of hnRNP A1 and hnRNP A2 reported previously, siRNAs of these two genes were transfected simultaneously. Briefly, exponentially growing A549 and Calu-6 cells were seeded into 6-well plates (2×10^5^ cells/well). The next day, 80 pmol of hnRNP A1 and hnRNP A2 (each 40 pmol), 40 pmol of SF2/ASF, or 40 pmol of PTB duplex RNAs (Beijing DNA-SYN Biotechnology Co., Beijing, China) were mixed with 6 µl Oligofectamine transfection reagent (Invitrogen) plus 250 µl of Opt-MEM medium (Invitrogen) for 20 min and were then added to cells, respectively. After transfection for 48 h, the cells were further transfected with 20 pmol of double-stranded RNA-Poly(I:C) (Sigma, St. Louis, MO) to activate signaling through the IRF-3 pathway. After Poly(I:C) stimulation for 24 h, total cellular RNA and protein were isolated for further analysis. For specific siRNA transfections, the mismatched siRNA transfection was used as mock-transfected control.

### Total RNA Isolation and Semi-Quantitative RT-PCR

Total cellular RNA was isolated using Trizol reagent (Invitrogen) according to the manufacturer's instructions. Approximately 3 µg of total RNA from each cell line was digested with RNase-free DNase I (Promega, Madison, WI) to remove DNA contamination and reverse transcribed into cDNA using the Superscript III System (Invitrogen). To examine the mRNA expression levels of target genes in cells, type-specific primer sets were designed and the corresponding PCR products were sequenced to confirm transcription accuracy. The primer sets, annealing temperature for amplification, and the length of PCR products are listed in Table 1. The PCR mixture consisted of 10 mM Tris-HCl pH 8.0, 50 mM KCl, 1.5 mM MgCl_2_, 200 pmol for each primer, 2 U Taq DNA polymerase (Promega), and a 1–2 µl of sample cDNA. The resulting fragments were subjected to electrophoresis on a 1.2–2.5% agarose gel and visualized with ethidium bromide staining. All PCR reactions were repeated with reproducible results. β-actin was used as internal control and total RNA extracted from the same cells without reverse transcription was used as negative control. To ensure that the PCR reactions fall within the linear range of amplification, the proper numbers of cycling for amplification of each control or target gene were examined (data not shown).

**Table 1:** Primers for semi-quantitative RT-PCR analysis.

Genes	Primers, 5′–3′	Annealing	Product
		(°C)	(bp)
*β-actin*		57	638
F	GGAGAAAATCTGGCACCACAC		
R	CGTACAGGTCTTTGCGGATGT		
*hnRNP A1*		56	612
F	TGCCTTTGTAACCTTTGACG		
R	ACTGTGCTTGGCTGAGTTCAC		
*hnRNP A2*		56	435
**F**	GATGGATATGGCAGTGGAC		
R	CTGTTACCTCTGGGCTCTCATC		
*SF2/ASF*		57	213
F	GAGATGGCACTGGTGTCGTG		
R	TGCGACTCCTGCTGTTGCTTC		
*PTB*		56	121
F	CACGCTGCACCTCTCCAACATC		
R	TGCCATCTTGCGGTCCTTCTG		
*IRF-3*		57	500 (FL)327 (I)155 (II)
F	AGCCTCGAGTTTGAGAGCTACC		
R	GGTATCAGAAGTACTGCCTCCAC		
*IFNβ*		60	110
F	AGCTGCAGCAGTTCCAGAAG		
R	AGTCTCATTCCAGCCAGTGC		
*IP-10*		58	222
F	GCATTCAAGGAGTACCTCT		
R	CCTTGCTAACTGCTTTCAG		

Products corresponding to IRF-3: FL, full-length IRF-3; I and II, two kinds of splicing variant (Type I and Type II).

### Western Blot Analysis

Cells were lysed for 30 min in lysis buffer (15 mM Tris-HCl pH 7.5, 150 mM NaCl, 0.1% Tween 20, and 1 mM DTT) supplemented with protease inhibitors and the solution was cleared by centrifugation at 12,000 rpm for 15 min. Total protein (25 µg) in 1×sodium dodecyl sulfate (SDS) sample buffer was separated by 10% SDS-PAGE and electro-transferred to nitrocellulose membrane (Amersham Pharmacia Biotech, Chicago, IL). The membranes were then blocked with 5% nonfat milk and probed with specific primary antibodies: anti-hnRNP A1 (Proteintech Group, Inc., Chicago, IL), anti-hnRNP A2B1 (Proteintech), anti-SF2/ASF (Santa Cruz Biotechnology, Santa Cruz, CA), anti-PTB (Proteintech), anti-Actin (I-19) (Santa Cruz), or anti-IRF-3 (Proteintech) (1∶1000 to 1∶3000 dilution), respectively. After washing with 0.2% Tween 20/PBS buffer three times, membranes were incubated with horseradish peroxidase-conjugated secondary antibody and visualized using the enhanced chemiluminescence system, ECL (GE Healthcare, Buckinghamshire, UK).

### Electrophoretic Mobility Shift Assay (EMSA)

The sequence of the intron 1 of IRF-3 pre-mRNA, upstream of exon 2 5′ splice site, contains UAGGGA sequence that is the same as the consensus hnRNP A1/A2 high affinity binding site identified by SELEX, UAGGG(A/U) [39]. By using ESE Finder program, other two binding sites (GGAAGGA) of SF2/ASF were identified immediately upstream of the exon 2 5′ splice site. To explore the possibility that hnRNP A1/A2 and SF2/ASF bind to these binding motifs, fusion proteins GST-hnRNP A1 and GST-SF2/ASF were expressed by IPTG induction and purified with Glutathione Sepharose 4B. Synthetic RNA oligonucleotides containing wild type binding motifs for hnRNP A1/A2 or SF2/ASF (wt-A1 or wt-SF2), the G3C mutant binding motif for hnRNP A1/A2 (mu-A1), or the G2C mutant binding motif for SF2/ASF (mu-SF2) were biotinylated using the Pierce RNA 3' End Biotinylation Kit (Thermo Scientific Pierce, Rockford, IL) following the manufacturer's instructions. The gel shift reactions were performed using the LightShift Chemiluminescent EMSA Kit (Pierce) according to the manufacturer's description. Briefly, 20 fmol of biotinylated wild type or mutant RNA probes were incubated with 50 ng of purified GST fusion proteins for 20 min at room temperature in a 20-µl binding reaction containing 2 µg tRNA. The EMSA reactions were electrophoresed on a 6% native PAGE, transferred to a positively charged nylon membrane (Amersham), and then cross-linked using a GS Gene Linker UV chamber (BioRad, Hercules, CA). Detection of biotinylated RNA was performed using stabilized streptavidin/horseradish peroxidase conjugate (Pierce) according to the manufacturer's instructions. To demonstrate the specificity of protein-RNA complex formation, purified GST protein was used for EMSA reaction and acted as negative control.

### IRF-3 Minigene Construction and Transfection

A wild type minigene containing the relevant IRF-3 genomic region from exon 1 to exon 4, and flanking regions was cloned into pcDNA3.0 vector (Invitrogen) using *Kpn* I and *Eco*R I cutting sites. The intron 1 of wt-IRF-3 minigene contains the putative binding sites for hnRNP A1/A2 (wt-A1) and SF2/ASF (wt-SF2). Minigenes with the G3C mutant binding site for hnRNP A1/A2 (mu-A1) or the G2C mutant binding site for SF2/ASF (mu-SF2) were generated through a two-step PCR overlap extension [40] using the wt-IRF-3 minigene construct as a template. All expression cassettes are under the control of the CMV promoter and a polyA signal. All the constructs were verified by sequencing. Wild type and mutated minigene vectors were transfected into A549 cells. Forty-eight hours after transfection, cells were collected and the relative expression levels of the IRF-3 isoforms were analyzed using RT-PCR.

### Isolation and Cultivation of Peripheral Blood Mononuclear Cells (PBMC) and Co-cultivation with A549 Cells

The study protocol and consent documents were approved by the Ethics and the Academic committees of Peking University First Hospital. Peripheral vein blood samples were obtained from healthy donors with informed consent. Isolation and cultivation of PBMC was performed as previously described [41]. Briefly, PBMC were isolated from peripheral blood using Histopaque®-1077 (Sigma) following the manufacturer's instructions. For cultivation of PBMC, cells were resuspended in RPMI 1640 supplemented with 5% heat-inactivated FBS (Invitrogen), 100 U/ml penicillin, 100 µg/ml streptomycin, 5 µg/ml Phytohemagglutinin (PHA) (Sigma) and 10 mM HEPES and seeded at 2×10^5^ cells/well in 6-well plates.

For subsequent co-cultivation with PBMC, A549 cells at 60–80% confluence were transfected with specific siRNAs as described above. At 48 h post-transfection, the cells were further transfected with Poly(I:C). Four hours later, the medium was changed to PBMC co-culture medium [RPMI 1640 supplemented with 10% heat-inactivated FBS, 100 U/ml penicillin, 100 µg/ml streptomycin, 1 µg/ml PHA, and 10 mM HEPES]. PBMC (5×10^6^ cells) were seeded into Transwell-Clear inserts (0.4 µm pore size, Costar Group, Washington, DC) and placed on top of the A549 cultures. Co-cultivation was performed for 72 h and culture medium was then collected for further analysis.

### Detection of IFNβ, CXCL10/IP-10, TNF-α, and IL-10 by Enzyme-Linked Immunosorbent Assay (ELISA)

Concentrations of IFNβ (Pierce), CXCL10/IP-10 (SABiosciences, Frederick, MD), tumor necrosis factor-alpha (TNF-α) (SABiosciences), and interleukin-10 (IL-10) (SABiosciences) in cell culture supernatants were determined by ELISA according to the manufacturers' instructions. The data were measured at 450 nm with a BioRad microplate reader.

### Immunohistochemical Staining

For immunohistochemistry analysis of the expression of hnRNP A1, hnRNP A2, SF2/ASF, and IRF-3, 63 paraffin-embedded human NSCLC tumor tissues, 39 adjacent non-tumor control tissues, and 26 bronchiectasis tissues were obtained from Peking University First Hospital, Beijing, China. This study was approved by both the Ethics and the Academic committees of Peking University First Hospital, and informed consent was obtained from each subject.

All tissues were paraffin-embedded and were stained using S-P immunohistochemical method. Briefly, the slides were deparaffinised in xylene, rehydrated in graded ethanol, and then treated with PBS containing 3% hydrogen dioxide to block endogenous peroxidase. After preincubating in 10% goat serum for non-specific binding block, slides were then incubated with specific primary antibodies: anti-hnRNP A1 (Proteintech), anti-hnRNP A2B1 (Proteintech), anti-SF2/ASF (Santa Cruz), or anti-IRF-3 (Proteintech) at 4°C overnight. After rinsing, sections were subsequently incubated with biotinylated immunoglobulins for 15 min and then with streptavidin-peroxidase conjugate for 15 min. The signals were developed with DAB-H_2_O_2_ solution. The slides were counterstained with 5% hematoxylin, and then examined by light microscopy. Sections without primary antibody treatment were used as negative control. The staining was scored on a scale from 0 to IV as follows: 0, less than 5% cells were stained; I, 5–25% cells were stained; II, 25–50% cells were stained; III, 50–75% cells were stained; and IV, more than 75% cells were stained. Scores II-IV were classified as positive, while scores 0 and I were negative.

### Statistical Analysis

SPSS 15.0 software (SPSS Inc, Chicago, IL) was used in determining statistical significance. The continuous variables from different groups were shown as mean ± S.D. and were compared using *t* test. Chi-square test was used to analyze significance for hnRNP A1, hnRNP A2, SF2/ASF, and IRF-3 expression between malignant and benign lung tissue groups studied. Values of *P*<0.05 were considered statistically significant.

## Results

### Knockdown of HnRNP A1/A2 or SF2/ASF in Human NSCLC Cells Increases Exclusion of Exons 2 and 3 of IRF-3 Gene

**Figure 1:**
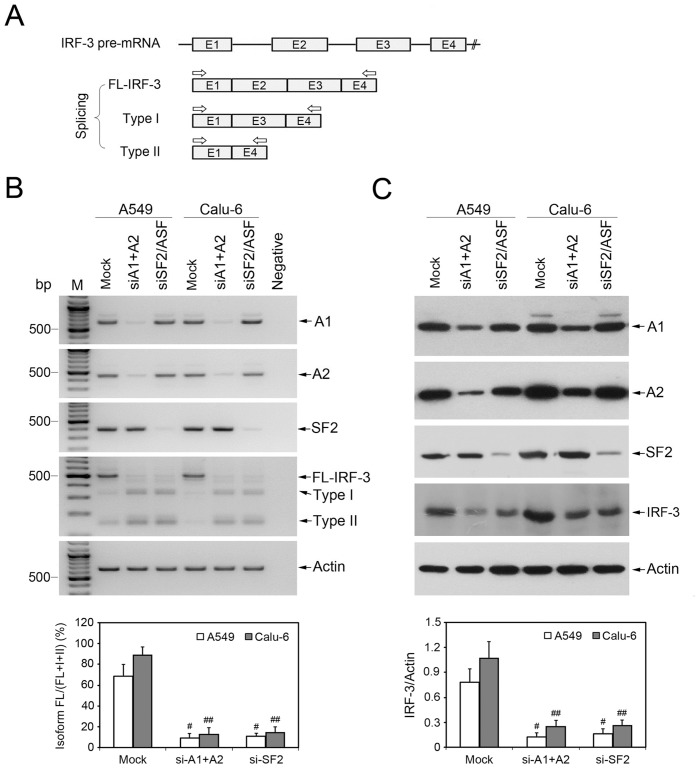
Depletion of hnRNP A1/A2 or SF2/ASF increases exclusion of exons 2 and 3 of IRF-3. (A) Schematic diagram showing the three splicing variants FL-IRF-3 (full-length IRF-3), Type I (only E2 exclusion) and Type II (both E2 and E3 exclusion) isoforms. E, exon. IRF-3 exons from 1 to 4 are numbered. The black solid line represents introns. The arrows above the transcripts show the location of specific primer sets designed for RT-PCR analysis of IRF-3 splicing variants. (B) Splicing factors hnRNP A1/A2 or SF2/ASF were depleted by specific siRNA transfection in human NSCLC cells A549 and Calu-6, respectively. A1, hnRNP A1; A2, hnRNP A2. The mismatched control siRNA was used for mock-transfected control. After siRNA transfection and subsequent Poly(I:C) stimulation, cells were harvested and semi-quantitative RT-PCR was performed to determine the impact of RNA interference on expression of target genes and IRF-3 splicing variants. Products corresponding to FL-IRF-3 and its two kinds of splicing variant (Type I and Type II) are indicated with arrows. For all reactions, total RNA extracted from A549 cells without reverse transcription was used as negative control. PCR products of FL-IRF-3, Type I and Type II isoforms were quantified by TotalLab Quant scanning. The graph indicates the ratio isoform FL/(FL+I+II) and the values are mean ± SD for n = 3 experiments. (C) Western blot analysis was performed with antibodies directed against the proteins indicated on the right. Protein bands of IRF-3 were also quantified and normalized to internal control Actin. The graph indicates the ratio IRF-3/Actin and the values are mean ± SD for n = 3 experiments. ^#^ *P* and ^##^ *P*<0.05 compared to mock-transfected A549 and Calu-6 cells, respectively.

To examine the possible effect of hnRNP A1/A2 and SF2/ASF on splicing regulation of IRF-3 gene, we performed siRNA-mediated depletion of hnRNP A1/A2 or SF2/ASF in NSCLC cells A549 and Calu-6. After siRNA transfection and subsequent Poly(I:C) stimulation of IRF-3 signaling pathway, total cellular RNA and protein were isolated and the abundance of hnRNP A1/A2 and SF2/ASF mRNAs and proteins was assessed by semi-quantitative RT-PCR and Western blot analysis, respectively. When compared with mock-transfected control extracts, the extracts from both A549 and Calu-6 cells transfected with specific siRNAs showed marked reduction in the expression levels of target genes (Figs. 1B and 1C). As expected, the SF2/ASF siRNA transfection did not affect the levels of hnRNP A1/A2, and vice versa.

Of the IRF-3 splicing variants, IRF-3b, -3c, -3d, and -3f lose exon 2 or both exon 2 and exon 3 [37]. To analyze change of alternative splicing of IRF-3 in siRNA-transfected cells, RT-PCR amplification of IRF-3 was performed using specific primer sets with forward primer located in exon 1 and reverse primer in exon 4. As shown in Figs. 1A and 1B, full-length IRF-3 (FL-IRF-3) and its two kinds of splicing variant (Type I: only exon 2 exclusion and Type II: both exon 2 and exon 3 exclusion) generated three bands: 500 bp (E1+2+3+4), 327 bp (E1+3+4), and 155 bp (E1+4) in length, respectively. As for A549 cells, combined depletion of hnRNP A1 and hnRNP A2 and single depletion of SF2/ASF both resulted in obvious decrease in FL-IRF-3 mRNA, from 67% to 9% or 11%, and concomitant increase in mRNAs of Type I and II isoforms (Fig. 1B). Similar results were obtained using Calu-6 cells, with knockdown of hnRNP A1/A2 or SF2/ASF resulting in decrease from 91% to 13% or 15% FL-IRF-3 mRNA (Fig. 1B). Single hnRNP A1 or hnRNP A2 depletion was also performed in NSCLC cells and showed no effect on IRF-3 splicing pattern (data not shown). Consistent with RT-PCR results, hnRNP A1/A2 or SF2/ASF depletion resulted in obvious decrease in expression levels of IRF-3 protein in both A549 and Calu-6 cells (Fig. 1C).

**Figure 2:**
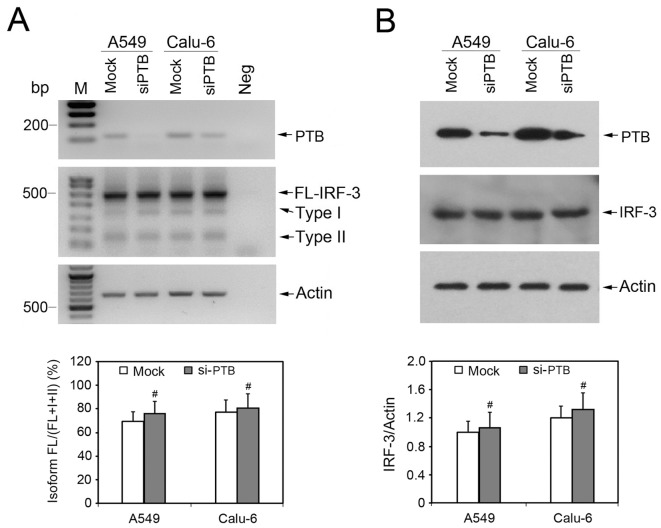
Depletion of PTB shows no effect on IRF-3 splicing pattern. Splicing factor PTB was depleted by specific siRNA transfection in human NSCLC cells A549 and Calu-6. The mismatched control siRNA was used as mock-transfected control. After siRNA transfection and subsequent Poly(I:C) stimulation, total cellular RNA and protein were collected and tested by semi-quantitative RT-PCR (A) and Western blot analysis (B) to examine the expression levels of target genes and IRF-3 splicing variants indicated on the right. For RT-PCR reactions, total RNA extracted from A549 cells without reverse transcription was used as negative control. For all RT-PCR and Western blot analysis, Actin was used as internal control.

To investigate more directly the specificity of hnRNP A1/2 or SF2/ASF depletion and exon skipping of IRF-3 pre-mRNA, we used siRNA to deplete PTB, another abundant negative regulator of alternative splicing. PTB depletion showed no effect on the splicing pattern of IRF-3 (Figs. 2A and 2B).

### HnRNP A1/A2 and SF2/ASF Bind Specifically to Sequences in IRF-3 Intron 1

**Figure 3:**
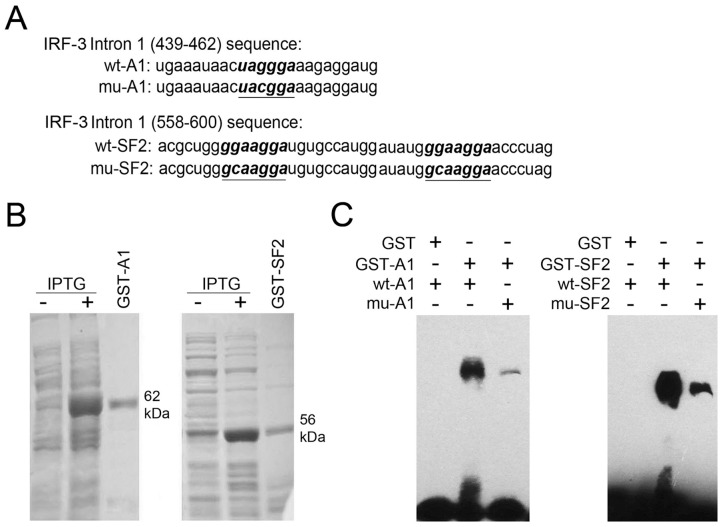
HnRNP A1/A2 and SF2/ASF bind specifically to sequences in IRF-3 intron 1. (A) Sequences of intron 1 (439–462) and intron 1 (558–600) of IRF-3. The putative binding sites for hnRNP A1/A2 (wt-A1) or SF2/ASF (wt-SF2) are indicated in bold italics. The G3C mutant binding site for hnRNP A1/A2 (mu-A1) and the G2C mutant binding site for SF2/ASF (mu-SF2) are underlined. (B) Fusion proteins GST-hnRNP A1 (GST-A1) and GST-SF2/ASF (GST-SF2) were expressed by IPTG induction and purified with Glutathione Sepharose 4B. (C) Twenty nanograms of each GST, GST-A1, and GST-SF2 protein were used for electrophoretic mobility shift assay with biotinylated RNA probes wt-A1 or wt-SF2 and their mutant derivatives.

Due to the identification of hnRNP A1/A2 and SF2/ASF binding motifs immediately upstream of the IRF-3 exon 2 5′ splice site (Fig. 3A), we further explored the possibility that hnRNP A1/A2 and SF2/ASF bind specifically to these binding motifs. Fusion proteins GST-hnRNP A1 and GST-SF2/ASF were expressed by IPTG induction and purified with Glutathione Sepharose 4B (Fig. 3B). The GST fusion proteins were incubated with biotinylated RNA probes containing the UAGGGA or GGAAGGA sequences of IRF-3 intron 1 and EMSA analysis was performed. As shown in Fig. 3C, clear shift bands were found with GST-hnRNP A1 and GST-SF2/ASF proteins. Further G3C mutation of the UAGGGA motif and G2C mutation of the GGAAGGA motif led to large decrease in hnRNP A1 and SF2/ASF binding. These data confirmed the presence of hnRNP A1/A2 and SF2/ASF binding sites upstream of exon 2 5′ splice site of IRF-3 pre-mRNA.

### IRF-3 Minigenes with Mutated HnRNP A1/A2 or SF2/ASF Binding Motifs Increase Exclusion of Exons 2 and 3

**Figure 4:**
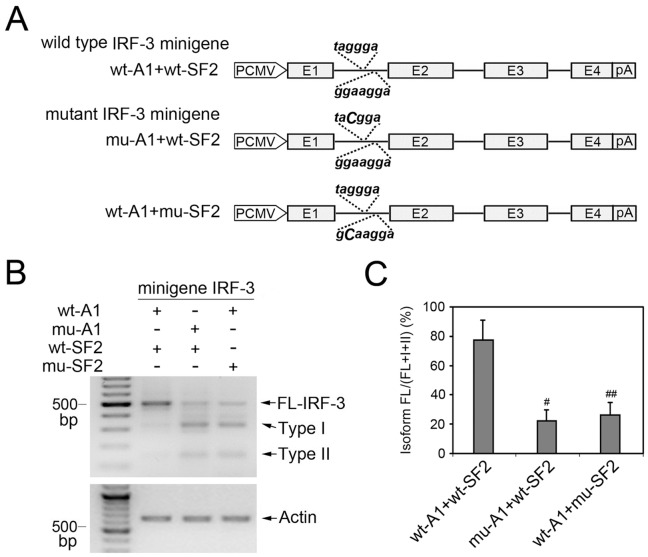
Minigene splicing assay of IRF-3. (A) The wild type (wt) and mutant (mu) versions of the IRF-3 minigene are shown. PCMV, promoter of the pcDNA3.0 vector. pA, polyA signal. IRF-3 exons from 1 to 4 are numbered. The black solid line represents introns. (B) Transient transfection of wt-IRF-3 or mu-IRF-3 minigenes was performed in A549 cells and the relative expression levels of the IRF-3 isoforms were measured by RT-PCR analysis. (C) The graph indicates the ratio isoform FL/(FL+I+II) and the values are mean ± SD for n = 3 experiments. ^#^ *P* and ^##^ *P*<0.05 compared to wt-IRF-3 minigene transfection.

To further explore the roles of hnRNP A1/A2 or SF2/ASF binding motifs in splicing regulation of IRF-3 gene, IRF-3 minigenes containing wild type or mutant hnRNP A1/A2 or SF2/ASF binding motifs were constructed and transfected into A549 cells (Figs. 4A and 4B). The relative expression levels of the IRF-3 isoforms were measured by RT-PCR analysis. Compared with wild type minigene transfection, the mutation of hnRNP A1/A2 or SF2/ASF binding sites resulted in obvious decrease in the ratio isoform FL/(FL+I+II), from 79% to 23% or 28% (both *P*<0.05), respectively, as shown in Figs. 4B and 4C.

### SiRNA-Mediated Depletion of HnRNP A1/A2 or SF2/ASF Reduces Expression of IRF-3 Downstream Effector Molecules IFNβ and IP-10

**Figure 5:**
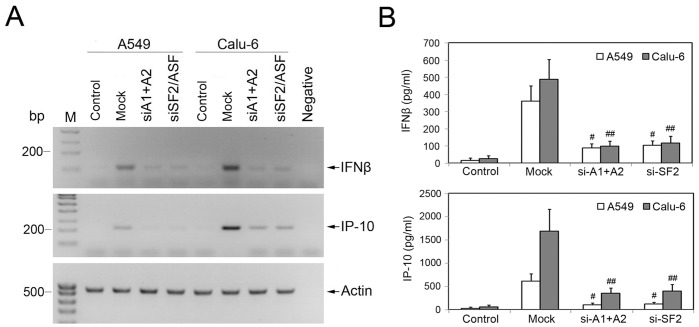
Depletion of hnRNP A1/A2 or SF2/ASF reduces IFNβ and IP-10 expression in human NSCLC cells. A549 and Calu-6 cells were performed specific siRNA-mediated knockdown of hnRNP A1/A2 or SF2/ASF. Mock, cells transfected with the mismatched control siRNA. Control, cells without specific or mock siRNA transfection and Poly(I:C) stimulation. (A) After siRNA transfection and subsequent Poly(I:C) stimulation, the mRNA expression of IFNβ and IP-10 genes was analyzed by semi-quantitative RT-PCR. Total RNA isolated from A549 cells without reverse transcription was used as negative control. (B) Secretion of IFNβ (top) and IP-10 (bottom) proteins was examined by ELISA assay. The value for each assay is presented as mean ± S.D. for three independent experiments. ^#^ *P* and ^##^ *P*<0.05 compared to mock-transfected A549 and Calu-6 cells, respectively.

To explore the impact of IRF-3 alternative splicing regulated by knockdown of hnRNP A1/A2 or SF2/ASF on expression of IRF-3-inducible genes, we isolated total RNA from A549 and Calu-6 cells after specific siRNA transfection and subsequent Poly(I:C) stimulation and performed semi-quantitative RT-PCR to analyze the expression levels of IFNβ and IP-10 genes. Moreover, the supernatant of cell culture was also collected and IFNβ and IP-10 proteins were examined by ELISA assay using anti-IFNβ or anti-IP-10 antibodies, respectively. As shown in Figs. 5A and 5B, A549 and Calu-6 cells without Poly(I:C) stimulation showed very low expression levels of IFNβ and IP-10, as compared to cells with Poly(I:C) stimulation. As expected, down-regulation of IFNβ and IP-10 genes in both mRNA and protein levels was paralleled by significantly decreased expression levels of IRF-3 protein in both A549 and Calu-6 cells transfected with hnRNP A1/A2 or SF2/ASF siRNA (Figs. 1C, 5A, and 5B).

### Knockdown of HnRNP A1/A2 or SF2/ASF by SiRNA Mediates Proinflammatory Effects as Detected in A549 Cell/PBMC Co-culture Experiments

**Figure 6:**
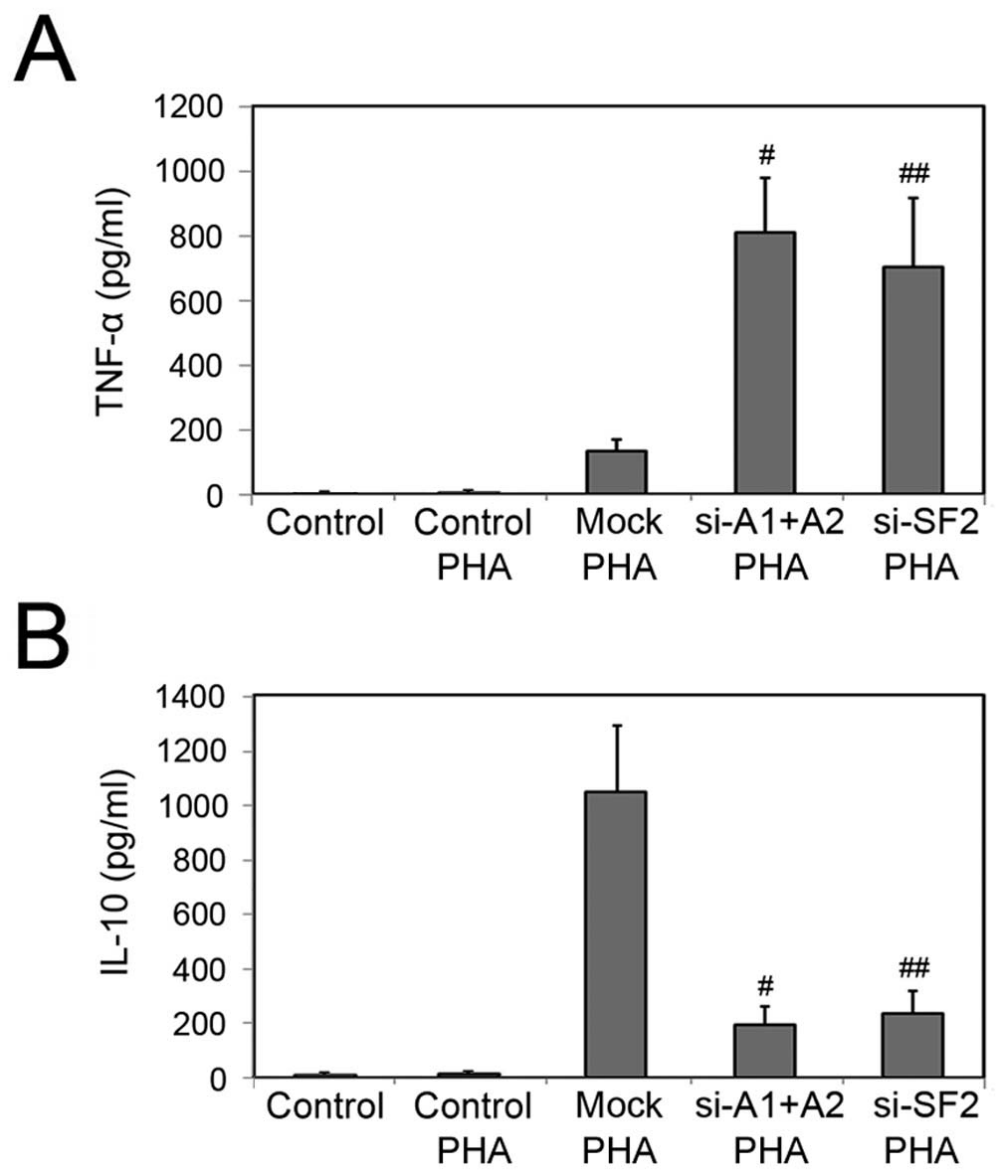
Depletion of hnRNP A1/A2 or SF2/ASF mediates proinflammatory effects as detected in A549 cell/PBMC co-cultures. To explore the overall regulatory potential of A549 cells with specific siRNA-mediated hnRNP A1/A2 or SF2/ASF depletion on inflammatory cytokine production, co-cultivation with human PBMC was performed in the presence of PHA. Control, A549 cells cultured without PBMC. Mock, A549 cells transfected with the mismatched control siRNA. The operational approach of A549 cell/PBMC co-culture was performed as described in Materials and Methods section. After 72 h, co-culture supernatants were collected and production of TNF-α (A) and IL-10 (B) was examined by ELISA assay. Data are shown as mean ± S.D. for three independent experiments. ^#^ *P* and ^##^ *P*<0.05 compared to co-cultures with mock-transfected cells.

To further examine the overall regulatory capacity of A549 cells with siRNA-mediated hnRNP A1/A2 or SF2/ASF depletion on inflammatory cytokine production, co-cultivation with freshly isolated human PBMC was performed in presence of PHA. By using PBMC, containing lymphocytes, dendritic cells, monocytes, and a few other cells (such as hematopoietic stem cells), we can imitate immune inflammatory cells within tumor microenvironment. As shown in Fig. 6A, pre-transfection of A549 cells by hnRNP A1/A2 or SF2/ASF siRNA obviously enhanced secretion of TNF-α by PBMC cells under the influence of PHA. However, PHA-induced release of IL-10 was significantly suppressed (Fig. 6B), suggesting a proinflammatory effect mediated by hnRNP A1/A2 or SF2/ASF siRNA in human NSCLC cells. In contrast, when cultured without PBMC, A549 cells exposed to Poly(I:C) did not display secretion of TNF-α or IL-10, irrespective of the presence or absence of PHA (Figs. 6A and 6B).

### HnRNP A1/A2, SF2/ASF, and IRF-3 Protein Levels are Elevated in Human NSCLC Tumor Tissues Compared with Non-Tumor Control Tissues

HnRNP A1/A2 and SF2/ASF have been reported to be up-regulated in multiple human cancers. To further illustrate the expression changes of these genes in human NSCLC tumor tissues compared with non-tumor control tissues, we tested hnRNP A1/A2, SF2/ASF, and IRF-3 expression by immunohistochemical staining of tissue sections from 63 NSCLC tumor tissues and 39 adjacent non-tumor control tissues as well as 26 bronchiectasis tissues.

**Figure 7:**
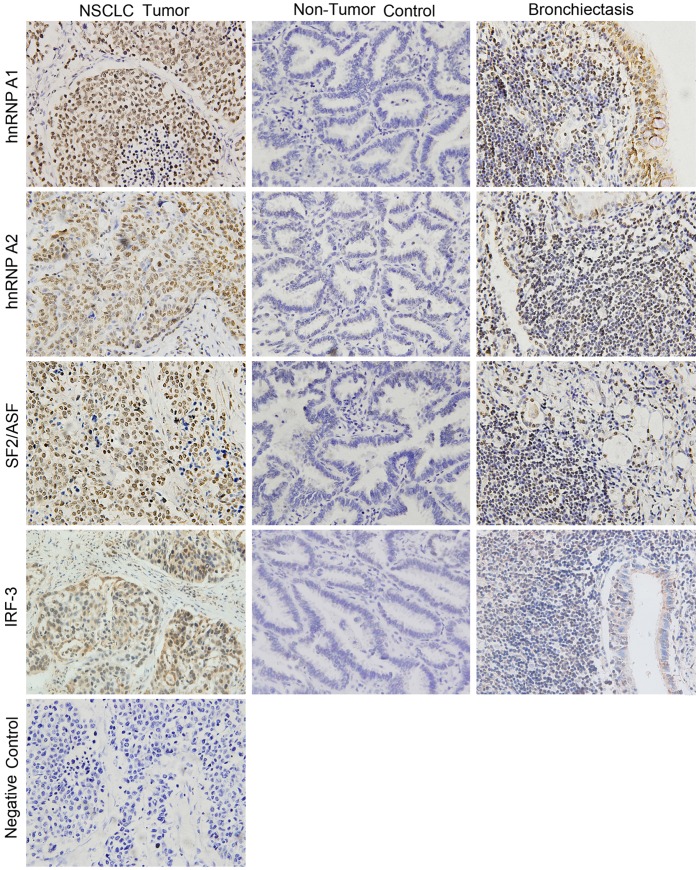
HnRNP A1/A2, SF2/ASF, and IRF-3 are over-expressed in human NSCLC tumor tissues. Representative images showing staining for hnRNP A1/A2, SF2/ASF, or IRF-3 with specific antibody of fixed lung sections of either NSCLC tumor tissue, non-tumor tissue, or bronchiectasis tissue. Positive hnRNP A1/A2 or SF2/ASF expression was shown as brown staining predominantly in the cell nucleus, while positive IRF-3 expression in both the cytoplasm and the nucleus. Section without antibody treatment was used as negative control. Original magnification, ×400.

The brown signals located predominantly in the nucleus represented positive staining for hnRNP A1, hnRNP A2, or SF2/ASF protein, whereas positive IRF-3 expression was shown in both the cytoplasm and the nucleus (Fig. 7). The degree of immune staining was scored as described in Materials and Methods. HnRNP A1/A2, SF2/ASF, and IRF-3 positivity in lung tissues was calculated and represented in Table 2. Among the lung tissues tested, 82.5% (52/63) NSCLC tumor tissues and 57.7% (15/26) bronchiectasis tissues were scored hnRNP A1 staining positive II and above, while 20.5% (8/39) non-tumor tissues were stained with the same degree. Additionally, hnRNP A2 and SF2/ASF showed the similar elevated expression levels in NSCLC tumor tissues and bronchiectasis tissues compared with non-tumor tissues. Positive rate of the IRF-3 protein in NSCLC tumor tissues (27/63, 42.9%) obviously increased in comparison with non-tumor tissues (5/39, 12.8%). Consistent with our findings in NSCLC cells, lung tissues with both hnRNP A1 and A2 negative expression presented significantly decreased positive rate of IRF-3 expression, as compared to tissues with hnRNP A1 or A2 positive expression (10.7% vs. 47.2%) (Table 3). Decreased expression of IRF-3 protein was also found in SF2/ASF negative lung tissues than SF2/ASF positive tissues (17.5% vs. 44.6%).

**Table 2:** Immunohistochemical analysis of hnRNP A1/A2, SF2/ASF, and IRF-3 in human lung tissues.

Gene symbol	Tissue types	Total number	Immunohistochemical staining score						
			IV	III	II	I	0	≥ II (%)	*P* Value
hnRNP A1	Non-tumor	39	0	0	8	12	19	8 (20.5)	
	NSCLC	63	18	23	11	8	3	52 (82.5)	<0.001
	Bronchiectasis	26	0	3	12	9	2	15 (57.7)	0.002
hnRNP A2	Non-tumor	39	0	0	5	8	26	5 (12.8)	
	NSCLC	63	7	16	19	15	6	42 (66.7)	<0.001
	Bronchiectasis	26	0	1	9	11	5	10 (38.5)	0.016
SF2/ASF	Non-tumor	39	0	0	7	11	21	7 (17.9)	
	NSCLC	63	12	17	16	13	5	45 (71.4)	<0.001
	Bronchiectasis	26	0	2	11	10	3	13 (50.0)	0.006
IRF-3	Non-tumor	39	0	0	5	7	27	5 (12.8)	
	NSCLC	63	4	9	14	20	16	27 (42.9)	0.001
	Bronchiectasis	26	0	0	8	8	10	8 (30.8)	0.076

NSCLC: human non-small cell lung cancer; Non-tumor: adjacent non-tumor control tissue;

Bronchiectasis: bronchiectasis tissue.

**Table 3:** Association of hnRNP A1/A2 or SF2/ASF expression with IRF-3 expression in human lung tissues (128).

	Total number	IRF-3 IHC no. (%)		*P* a Value
		≥ II (%)	< II (%)	
hnRNP A1/A2				<0.001#
Positive	72	34 (47.2)	38 (52.8)	
Negative	56	6 (10.7)	50 (89.3)	
SF2/ASF				0.001##
Positive	65	29 (44.6)	36 (55.4)	
Negative	63	11 (17.5)	52 (82.5)	

a Two-sided χ^2^ test,

# χ^2^ = 19.542,

## χ^2^ = 10.981.

## Discussion

IRF-3 is a unique member of the IRF family and plays critical roles in viral and microbial defense [25]–[31]. Alternative splicing of IRF-3 pre-mRNA has been shown to be an important mechanism for the functional regulation of IRF-3. IRF-3a, as the originally characterized IRF-3 splicing variant, provides a good example demonstrating how structural alteration may turn a protein into an antagonist of its normal counterpart. IRF-3a is generated by alternative utilization of an acceptor/donor splice site at the end of the IRF-3-specific exon 2 and has been reported to inhibit the transactivation potential of IRF-3 and decrease the expression of endogenous IFNβ [35], [36]. With the exception of IRF-3a, we recently identified five novel splicing variants of IRF-3 from human cells and tissues, named IRF-3b to -3f [37]. Of these novel splicing variants, only IRF-3e retained the original start codon in exon 2. Due to deletion of exon 2 in IRF-3d and -3f as well as deletion of both exon 2 and exon 3 in IRF-3b and -3c, a novel start codon was predicated to occur in exon 5 and thereby resulted in truncated proteins. As reported in our previous study, different patterns of functional domain deletion caused by exon exclusion affected the structure of IRF-3 and thereby changed the transactivation activities on target promoters as well as their binding capacities and the subcellular localization of these splicing variants.

In the present study, hnRNP A1/A2 binding site (UAGGGA) and SF2/ASF binding site (GGAAGGA) were identified in IRF-3 intron 1 by using sequence analysis and ESE Finder program. Subsequent RNA binding assays using purified GST fusion proteins (GST-hnRNP A1 or GST-SF2/ASF) and RNA probes containing wild type or mutant hnRNP A1/A2 or SF2/ASF binding sites confirmed the presence of hnRNP A1/A2 and SF2/ASF binding motifs in IRF-3 intron 1. To explore whether these splicing regulators are involved in exclusion or inclusion of exons 2 and 3, we used siRNA to deplete hnRNP A1/A2 or SF2/ASF in human NSCLC cells A549 and Calu-6 and performed semi-quantitative RT-PCR to analyze change of alternative splicing of IRF-3 using consensus primers located in exon 1 and exon 4. As shown in Figs. 1A and 1B, FL-IRF-3 and its two kinds of splicing variant (Type I and Type II) generated three bands. Knockdown of hnRNP A1/A2 or SF2/ASF in both A549 and Calu-6 cells resulted in significant change of IRF-3 splicing pattern-obvious decrease in FL-IRF-3 mRNA and concomitant increase in mRNAs of Type I and II isoforms. Consistent with RT-PCR results, hnRNP A1/A2 or SF2/ASF depletion resulted in obvious decrease in expression level of IRF-3 protein in both A549 and Calu-6 cells. Further minigene splicing assay showed that IRF-3 minigenes with mutated hnRNP A1/A2 or SF2/ASF binding motif increased exclusion of exons 2 and 3. Further depletion of another factor of hnRNP family, PTB, showed no effect on the splicing pattern of IRF-3, suggesting that regulation of IRF-3 splicing by hnRNP A1/A2 or SF2/ASF is substrate-specific, and not the result of general regulatory properties of these proteins. Taken together, these results established direct and specific correlation between hnRNP A1/A2 or SF2/ASF binding to the relevant binding motifs in IRF-3 intron 1 and exclusion of exons 2 and 3.

Both IFNβ and IP-10 are determined as IRF-3-dependent genes and alterations in their expression levels have been associated with inflammatory diseases, immune dysfunction, and tumor development [27]-[31]. We next examined the influence of IRF-3 alternative splicing regulated by depletion of hnRNP A1/A2 or SF2/ASF on expression of IFNβ and IP-10 genes in NSCLC cells using semi-quantitative RT-PCR and ELISA approaches. Our data showed that siRNA-mediated depletion of hnRNP A1/A2 or SF2/ASF reduced expression of IFNβ and IP-10 genes in both A549 and Calu-6 cells, agreed with the decreased levels of IRF-3 protein. IFNβ has been reported to act in an autocrine or paracrine manner to amplify the downstream cascades of IFN-stimulated genes expression. To observe overall regulatory potential of knockdown of hnRNP A1/A2 or SF2/ASF on cytokine production, co-cultivation with freshly isolated human PBMC was performed. PBMC contain myriad immune inflammatory cells, including lymphocytes, dendritic cells, and monocytes, which can produce various kinds of cytokines. Our data showed that depletion of hnRNP A1/A2 or SF2/ASF in A549 cells reinforced PHA-induced TNF-α release by PBMC cells but suppressed that of IL-10 in medium of A549/PBMC co-cultivation.

Both TNF-α and IL-10 play important roles in immunoregulation. TNF-α is a multifunctional proinflammatory cytokine secreted predominantly by monocytes/macrophages [41], [42]. Dysregulation of TNF-α has been reported to play a critical role in orchestrating inflammatory processes by switching on a TNF-α-dependent cytokine cascade [43]. On the contrary, IL-10, as an anti-inflammatory cytokine, reduces expression levels of circulating TNF-α and IL-6 and is indispensable condition for development and function of the regulatory T cells-Tregs, which are important immunosuppressive inflammatory cells involved in suppressing anti-tumor immune responses and developing T cell tolerance [44], [45]. Consistent with previous report that adenovirus-mediated IRF-3 transgene expression changed the microglial cytokine profile from a proinflammatory phenotype to an anti-inflammatory phenotype [46], our data in human NSCLC cells showed that specific knockdown for hnRNP A1/A2 or SF2/ASF could contribute to forming a proinflammatory cytokine milieu through regulating IRF-3 splicing pattern and expression of downstream effector molecules.

Since silencing hnRNP A1/A2 or SF2/ASF in human NSCLC cells reduced expression levels of IRF-3 protein, we next examined the expression of hnRNP A1/A2, SF2/ASF, and IRF-3 in 63 NSCLC tumor tissues, 39 non-tumor control tissues, and 26 bronchiectasis tissues. Our immunohistochemical staining results showed that the expression levels of hnRNP A1/A2 and SF2/ASF were significantly elevated in human NSCLC tumor tissues and bronchiectasis tissues compared with non-tumor control tissues, indicating a correlation of hnRNP A1/A2 and SF2/ASF over-expression with inflammatory diseases and tumorigenesis of human NSCLC. Elevated IRF-3 protein expression was detected in NSCLC tumor tissues compared with non-tumor control tissues (42.9% vs. 12.8%, *P* = 0.001). Increased positive rate of IRF-3 protein was also found in bronchiectasis tissues compared with non-tumor control tissues, but showed no significant difference (30.8% vs. 12.8%, *P* = 0.076). We further investigated the association of hnRNP A1/A2 and SF2/ASF expression with IRF-3 expression in all human lung tissues tested. Decreased expression of IRF-3 protein was found in hnRNP A1/A2 or SF2/ASF negative lung tissues (10.7% or 17.5%) than hnRNP A1/A2 or SF2/ASF positive tissues (47.2% or 44.6%), respectively. Our immunohistochemical staining results suggested significant correlation between increased positive rate of IRF-3 protein and elevated hnRNP A1/A2 or SF2/ASF expression in human NSCLC tissues. Although ectopic expression of IRF-3 was previously shown to suppress the growth of HeLa cervical cancer cells [47] and B16 melanoma tumors [48], thus far, the involvement of IRF-3 expression in human cancer tissues has not been studied extensively. Takayuki Tokunaga et al. reported that IRF-3 expression was positive in 82% (41/50) human NSCLC tissues and 22.2% (10/45) NSCLC tissues possessed sequence variants of IRF-3 coding region with amino acid changes (S175R, A208D, and S427T) [49]. Takayuki Tokunaga et al. further revealed that the D208 variant presented significantly lower transcriptional activity, as compared to the wild type IRF-3. Other studies also suggested the association of IRF-3 (S427T) with increased risk for HPV infection [50] or for colon cancer [51]. To further elucidate the association of IRF-3 expression with NSCLC tumorigenesis, IRF-3 expression levels and gene structures in expanded paired NSCLC tumor and normal control tissues merit further investigation.

Tumor-related immunity and inflammation, belonging to biological characteristics of tumor, participate in cancer progression. So far, there have been numerous evidences for pro-tumoral or anti-tumoral activity of inflammation. On the one hand, chronic inflammation has been reported to stimulate cellular proliferation, invasion, angiogenesis, and metastasis and inhibit apoptosis through producing bioactive molecules such as cytokines, chemokines, and growth factors [52]-[54]. On the other hand, some studies have revealed that by recruiting immunosuppressive inflammatory cells such as Tregs and myeloid-derived suppressor cells (MDSCs), cancer cells can contribute to immunosuppressive tumor microenvironment and eventually promote tumor progression [55]–[57].

In this work, we demonstrated for the first time that silencing hnRNP A1/A2 or SF2/ASF in human NSCLC cells increased exclusion of exons 2 and 3 of IRF-3 gene and reduced expression levels of IRF-3 protein and IRF-3 downstream effector molecules IFNβ and IP-10, which further influence cytokine production and contribute to forming a proinflammation cytokine milieu in human NSCLC/PBMC co-cultures. Interestingly, some inflammatory mediators, such as CXCL12 and TGF-β1, have been reported to be able to modify expression pattern of hnRNP A2/B1 [58], [59]. Then the influence of IRF-3 alternative splicing regulated by hnRNP A1/A2 and SF2/ASF on immunomodulatory functions of human tumor cells or immune cells justifies more extensive research.
